# GROWING AN ONLINE GERONTOLOGY MINOR AT THE UNIVERSITY OF UTAH

**DOI:** 10.1093/geroni/igz038.2286

**Published:** 2019-11-08

**Authors:** Jacqueline Eaton

**Affiliations:** University of Utah, Salt Lake City, Utah, United States

## Abstract

The Gerontology Interdisciplinary Program at the University of Utah has a long history of offering certificates (48 years) and a Master of Science degree (25 years). Relatively new to our program is the minor in gerontology, first available in 2013. This paper will describe efforts to grow this program, barriers to enrollment, and plans for expansion. In 2018, the program developed a targeted strategy to increase student awareness of and enrollment in the minor. First, we focused on newly enrolled pre-nursing students through new student orientation presentations and registration support. The introductory course within the minor jumped in enrollment from 6 students in Fall of 2017 to 40 in Fall 2018. Students taking courses in the minor received follow-up support and instructions mid-semester to ease matriculation. Enrollment in the minor increased by 140%. Next, we plan to apply these strategies to a broader number of colleges and departments throughout campus.

